# 3D Convolutional Neural Networks Initialized from Pretrained 2D Convolutional Neural Networks for Classification of Industrial Parts

**DOI:** 10.3390/s21041078

**Published:** 2021-02-04

**Authors:** Ibon Merino, Jon Azpiazu, Anthony Remazeilles, Basilio Sierra

**Affiliations:** 1 TECNALIA, Basque Research and Technology Alliance (BRTA), Mikeletegi Pasealekua 7, 20009 Donostia-San Sebastián, Spain; jon.azpiazu@tecnalia.com (J.A.); anthony.remazeilles@tecnalia.com (A.R.); 2 Robotics and Autonomous Systems Group, Universidad del País Vasco/Euskal Herriko Unibertsitatea, 48940 Basque, Spain; b.sierra@ehu.eus

**Keywords:** computer vision, deep learning, transfer learning, object recognition

## Abstract

Deep learning methods have been successfully applied to image processing, mainly using 2D vision sensors. Recently, the rise of depth cameras and other similar 3D sensors has opened the field for new perception techniques. Nevertheless, 3D convolutional neural networks perform slightly worse than other 3D deep learning methods, and even worse than their 2D version. In this paper, we propose to improve 3D deep learning results by transferring the pretrained weights learned in 2D networks to their corresponding 3D version. Using an industrial object recognition context, we have analyzed different combinations of 3D convolutional networks (VGG16, ResNet, Inception ResNet, and EfficientNet), comparing the recognition accuracy. The highest accuracy is obtained with EfficientNetB0 using extrusion with an accuracy of 0.9217, which gives comparable results to state-of-the art methods. We also observed that the transfer approach enabled to improve the accuracy of the Inception ResNet 3D version up to 18% with respect to the score of the 3D approach alone.

## 1. Introduction

Industrial processes are continuously changing and now digitalization and smart automation are the main focuses to improve performance and productivity of the industrial plants. Robotics, combined with computer science techniques, such as machine learning, have boosted exponentially the production and security. This industrial revolution has been named *Industry 4.0*. Many fields have been integrated to this paradigm. One of them is computer vision.

Computer vision is a sub-field of machine learning consisting in acquiring, processing, analyzing and understanding images of the real world in order to generate information that a computer can deal with. As in all artificial intelligence fields, deep learning techniques are extensively used nowadays in computer vision. Deep learning approaches usually need a big dataset to obtain significant results. In order to meet this requirement, some techniques use deep learning nets that have been trained with huge datasets and transfer that learned knowledge to smaller or different datasets. Those techniques are called transfer learning [1,2].

In industrial application, and particularly in SMEs, or when small production batches are targeted, making huge datasets can be too expensive and arduous. In addition, objects to be recognized can be small and uncommon. Reducing the number of images needed for training is critical in this context. Using transfer learning methods can help to reduce computing time and the minimum dataset size that is needed to obtain significant results.

In parallel, in recent years, 3D cameras are gaining more and more popularity, specially in robotic applications. Working with 3D data is a relatively new paradigm that 2D convolutional networks cannot handle so easily. Therefore, new deep learning methods have been designed to deal with this paradigm [3,4], like the 3D convolutional networks.

Our proposal is a transfer learning technique that relies on using 2D features learned by 2D convolutional nets to train a 3D convolutional net.

The rest of this paper is organized as follows: Section 2 outlines the related works, Section 3 details the proposed approach, Section 4 describes the training phase of the network, Section 5 shows the experimental results obtained, and Section 6 summarizes the conclusions.

## 2. Related Works

Recent improvements in computing power and the rapid development of more affordable 3D sensors, have opened a new paradigm where 3D data, such as point clouds, are providing better understanding of the environment. Even if some advances have been done in deep learning on point clouds, this is still an underdeveloped field compared to 2D deep learning [3].

Dealing with 3D data in deep learning opens many new fronts. For example, 3D data is difficult to label, so that a significant time is required to label training data. Therefore, usually, the size of the training set for 3D approaches is notably smaller than the one used with 2D techniques.

Many different Convolutional Neural Networks (CNN) have been possible and gained a great success due to the large amount of public image repositories, such ImageNet [5,6] and high-performance computing systems, like GPUs.

Two-dimensional CNN have been widely studied, and there are many successful methods, but 3D CNN still need more research, as we will show in the next sections.

### 2.1. 2D CNN

The most important deep learning architectures are identified through the ImageNet Large Scale Visual Recognition Competition (ILSVRC) [6]. One of the first ones winning this competition is Alexnet [5]. ZFNet [7] and OverFeat [8] followed Alexnet, improving the results they obtain for the ImageNet dataset.

Understanding of convolutional layers is improved by Reference [7], thanks to their visualization. The following architectures focused on extracting features on low spatial resolutions. One of them is VGG [9], which is still being used as a base to many other architectures because of its simple and homogeneous topology. VGG scored the second place in the ILSVRC 2014. The first place was achieved by GoogLeNet [10], also known as Inception Network. This network was an improvement of the AlexNet, reducing the number of parameters while being much deeper. They introduced the Inception module, which enabled to recognize patterns of different sizes within the same layer, concurrently performing several convolutions of different receptive fields and combining the results.

Another influential architecture was introduced by Reference [11] named the Residual blocks. The architecture called ResNet introduced those Residual blocks which include a skip connection on a convolution block that is merged by summation with the output of that block. This network won the ILSVRC 2015 localization and classification contests and also the COCO detection and segmentation challenges [12].

A modification of the GoogLeNet called Inception-v4 [13] included an improvement on the inception module and three different kinds of inception modules. In addition, this paper also presents a combination of the inception module with the residual connection, named Inception-ResNet, resulting in a more efficient network. Another network similar to the previous one, the ResNeXts [14], achieved the second place in the 2016 ILSVRC classification challenge. The first place in classification, localization, and detection challenges was achieved by ResNet101, Inception-v4, and Inception-ResNet-v1, respectively.

Due to the success of the Inception and Residual modules, many subsequent networks have been derived from them. For example, DenseNets [15] combine the output of the residual connection and the output of the residual block by depth wise filter concatenation. The 2017 ILSVRC localization challenge’s first place and the top 3 in classification and detection categories were won by Dual Path Network (DPN) [16], a network that combines the architectures of DenseNets and ResNet.

Since previous networks focus on achieving the highest possible accuracy, they are not prepared for real-time applications with restricted hardware, like mobile platforms. MobileNets [17] tackles this problem by replacing standard convolutions with Depthwise Separable Convolutions.

Recently, Reference [18] proposed a novel scaling method that uniformly scales network’s depth, width, and resolution, obtaining a new family of models called EfficientNet. This family achieves much better accuracy with a 6.1× gain factor in computation time and a 8.4× factor in size reduction compared to previous ConvNets.

### 2.2. 3D CNN

Some researchers have taken advantage of the fact that 2D deep learning is more mature than 3D deep learning, trying to obtain a solution to 3D based on 2D deep learning.

Recently, the arrival of RGB-D sensors, such as the Microsoft’s Kinect or the Intel’s Realsense, has enabled to acquire at a low cost 3D information. These sensors provide a 2D color image (RGB), along with a depth map (D), which provides the 3-dimensional information. Since both RGB and D are 2D images, 2D deep learning methods can be adapted to receive as input two images instead of one. Even if this representation is quite simple, they are effective for different tasks, such as human pose regression [19], 6D pose estimation [20], or object detection [21].

Despite representing 3D data, RGB-D images are composed by 2D data and no transformation is needed. One possible transformation as proposed in References [22,23], consists of projecting the 3D data into another 2D space while keeping some of the original 3D shape key properties.

To keep 3D data without transforming it to 2D, some works, like References [24,25], propose a Voxel-based method. Voxels are used to describe how the 3D object is distributed in the three dimensions of the space. This representation is not always the best option since it stores both the occupied and non-occupied parts of the scene. Voxel-based methods are not recommended for high-resolution data since they store a huge unnecessary amount of data. To deal with this problem, octree-based methods with varying sized voxels [26,27] are proposed.

In order to reduce the number of parameters, which is too high in voxel-based methods, some methods propose point-based methods that include point cloud as an unordered set of points as input [28,29].

Our proposal changes this perspective. We adapt the 2D deep learning architecture to 3D and transform the weights from 2D to 3D as initial weights for the newly generated 3D Convolutional model. This approach makes it possible to leverage on existing nets trained on 2D data and apply them on 3D data while maintaining the original data structure.

## 3. Proposed Approach

**Figure 1:**
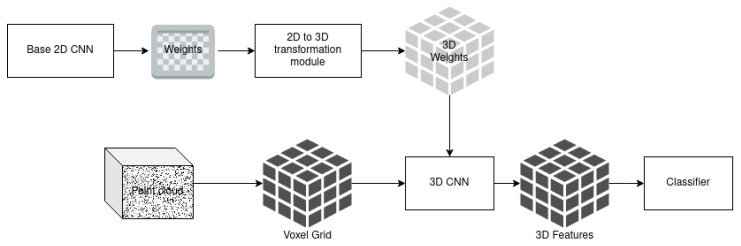
Overall architecture of the proposed method.

Due to the great success of 2D CNN in computer vision, our proposal uses those nets as the base to train a 3D CNN for classification. Figure 1 shows the overview of the proposed architecture. First, the weights of a pre-trained 2D CNN are transformed to 3D to be, therefore, used as the weights of the 3D CNN. The input point cloud is discretized by computing a voxel grid. That grid is the input tensor to the 3D CNN, which is an adapted form of the 2D CNN using 3D layers instead of 2D layers. That 3D CNN computes the 3D features that are then passed on to the classifier.

The following subsections explains the different modules of the architecture.

### 3.1. 2D to 3D Transformations

CNN weights can be represented as 2D matrices. Thus, we need to transform a 2D matrix into a 3D tensor, i.e., map the function M(x,y)=(r,g,b) to T(x,y,z)=(r,g,b), where x,y∈N, and r,g,b∈R. For each value of *x* and *y* of the 2D matrix and each of the new possible *z* values the transformation function is: $h\left(x,y,z,M\left(x,y\right)\right)=T\left(x^{\prime },y^{\prime },z^{\prime }\right)=\left(r,g,b\right)$. We have proposed 2 different transformation functions, the extrusion and the rotation.

#### 3.1.1. Extrusion

Extrusion of the plane consists of filling the tensor copying the RGB values along one axis. Given a matrix *M* of size (W×H) and the resulting tensor *T* of size (W×H×D) and the fact that we use inputs that have all the dimensions of the same length, this is, W=H=D, the Extrusion mapping is defined as:

∀x,y,z≤W:T(x,y,z)=M(x,y).

The extrusion can be done along the three main axes:

- Z axis: T(x,y,z)=M(x,y),
- Y Axis: T(x,z,y)=M(x,y),
- X Axis: T(z,x,y)=M(x,y).

**Figure 2:**
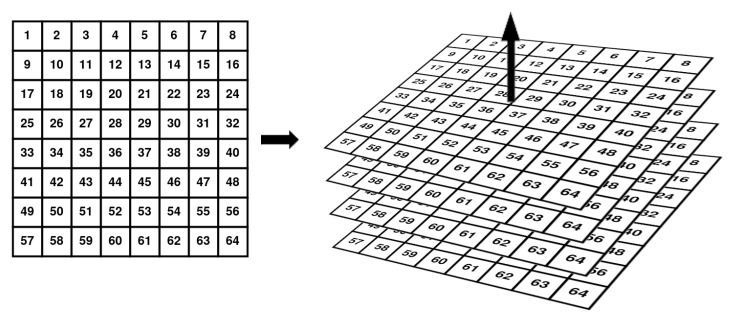
Extrusion along Z axis of a 2D matrix to generate a 3D tensor.

Figure 2 shows how the extrusion along the Z axis is done for a matrix.

#### 3.1.2. Rotation

To add curvature to the 2D weights, a rotation from 0 to 90 degrees with respect to the Z axis is applied to the 2D weights. The mapping from 2D to 3D is defined as:

$$T\left(x,y,z\right)=M(x,min\left(⎣\sqrt{y^{2}+z^{2}}⎦,W\right)).$$

**Figure 3:**
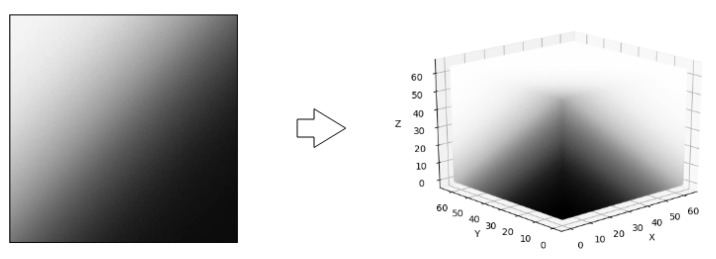
Rotation on Z axis of a 2D matrix to generate a 3D tensor.

Figure 3 shows an example of the 2D to 3D rotation transformation.

### 3.2. Discretization of the Point Cloud

**Figure 4:**
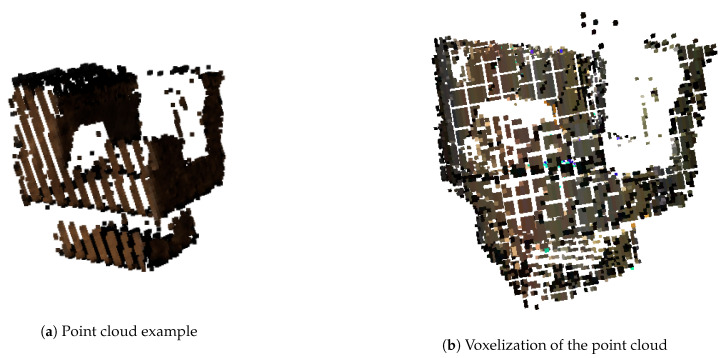
Transformation from a point cloud to a voxel grid.

The input of the architecture is a point cloud. In order to use a CNN, we have to discretize/sample the point cloud to a gridded structure (tensor). The representation used is the voxel grid, in which a voxel is the three-dimensional equivalent of a pixel. This method generates a three-dimensional grid of shape (nx, ny, nz), where each point of the point cloud is assigned to a voxel. If more than one point is assigned to the same cell, an interpolation is used to calculate the RGB value of that voxel. As an illustration, Figure 4a shows a point cloud and Figure 4b presents its voxelization. The number of voxels on each dimension (nx, ny, nz) depends on the architecture used, and it is explained in the subsection of each architecture.

### 3.3. Feature Extractor CNN

The most used CNN feature extractors in the literature are ResNet, Inception, VGG16, Xception, and Inception-ResNet v2. All of them can be transformed to 3D CNN by replacing the 2D convolutional layers with 3D convolutional layers and 2D MaxPooling layers with 3D MaxPooling layers. The weights of those nets trained with ImageNet are transformed to 3D to train the 3D version of those nets. The structure of each of the nets are described in the following subsections.

#### 3.3.1. VGG16

**Figure 5:**
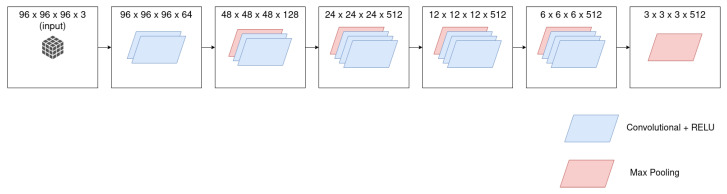
VGG16 architecture.

The 2D version of the VGG16 requires as input a 224×224 RGB image. In the 3D case, the input is a 96×96×96×3 (RGB) tensor. Figure 5 shows the architecture of the 3D version of the VGG16 feature extractor. This is composed of 2 Convolutional layers of 64 filters, MaxPooling layer, 2 Convolutional layers of 128 filters, MaxPooling layer, 3 Convolutional layers of 512 filters, MaxPooling layer, 3 Convolutional layers of 512 filters, and a MaxPooling layer. The convolution receptive field is 3 × 3, the stride is fixed to 1, padding to 1, and MaxPooling is performed over a 2 × 2 × 2 window with a stride of 2. The 2D Convolutional layers have been replaced by 3D Convolutional layers and 2D MaxPooling by 3D MaxPooling.

#### 3.3.2. ResNet

The Residual Neural Network’s (or ResNet) main contribution is what is called *skip connections*, which allows output from previous layers to bypass the next layers in order to propagate residual information (Residual Blocks). The ResNet can be tuned by changing the number of blocks that each group has. Table 1 presents different configurations of the 3D ResNet that we have used in the experiments.

**Figure 6:**
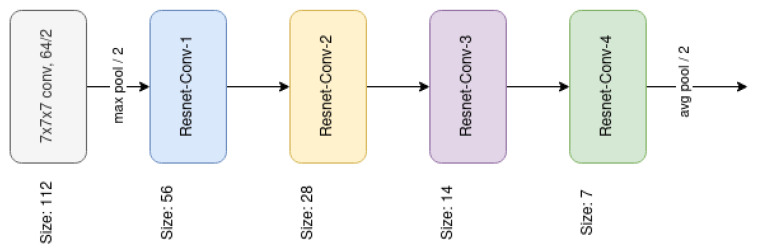
ResNet architecture. Building blocks are shown in brackets.

**Figure 7:**
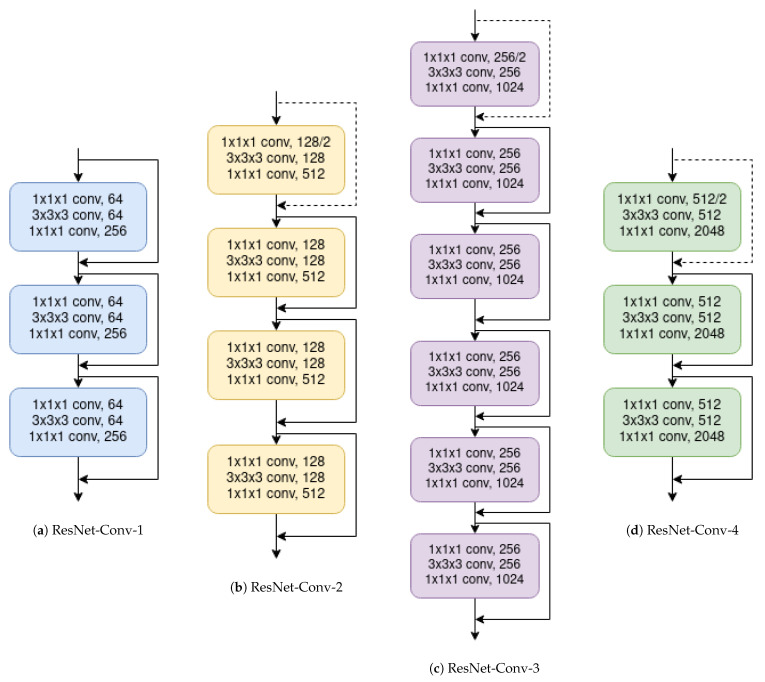
Building blocks of the Resnet50.

Figure 6 shows the architecture of the ResNet50. Figure 7a–d are the building blocks that form the ResNet50. In order to transform this network to 3D, Convolutional and MaxPooling layers have been changed by their respective 3D version. AveragePooling also has its 3D version. The input 2D image size is 224×224, while the 3D input tensor is 96×96×96.

#### 3.3.3. Inception-ResNet v2

**Figure 8:**
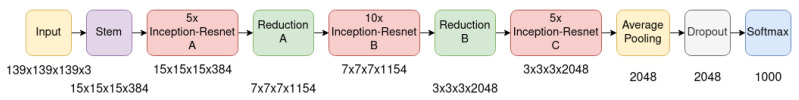
Inception-ResNet-v2 architecture.

This network combines the idea of the two previous networks: Inception blocks and Residual blocks. Authors of this architecture [13] propose two different versions: v1 and v2. Inception-ResNet-v2 is a wider version which is similar to Inception-v4 but adding Residual blocks. Figure 8 shows the 3D architecture of this second version of the network. The Inception-ResNet blocks, the Reduction blocks, and Stem block are described in Reference [13].

To adapt this 2D Convolutional Network to 3D, we have changed the 2D Convolutional layers to 3D Convolutional layers, the 2D Average Pooling layers to 3D Average Pooling layers, the 2D MaxPooling layers to 3D MaxPooling layers, and we use 3D vector as stride instead of 2D vector and 3D kernel size instead of 2D kernel size. The input size of this architecture is 139×139×139×3 (RGB) instead of 299×299×3 so that the resulting outputs of the blocks are 15×15×15×384, 7×7×7×1154, and 3×3×3×2048, respectively, for the Stem block, the Reduction A block, and the Reduction B block.

#### 3.3.4. EfficientNet

Given a base network, it is typical to scale its depth, width and resolution in order to improve accuracy. In Reference [18], researchers stated that *“Scaling up any dimension of network width, depth or resolution improves accuracy, but the accuracy gain diminishes for bigger models”* and that *“In order to pursue better accuracy and efficiency, it is critical to balance all dimensions of network width, depth and resolution during ConvNet scaling”*. Consequently, they proposed a compound scaling method that uses a compound coefficient to uniformly scale the 3 components.

**Figure 9:**
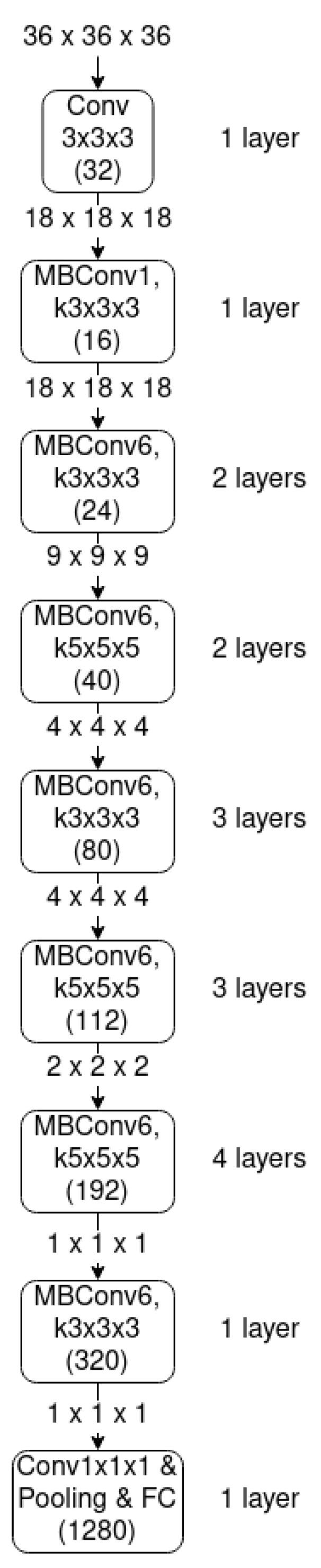
EfficientNet base architecture (EfficientNetB0).

The base network from which they start scaling is called EfficientNet-B0 (see Figure 9). This architecture is similar to the MnasNet [30]. The main building block is a mobile inverted bottleneck MBConv [30,31], to which is added a squeeze-and-excitation optimization [32]. This base network is scaled up applying the compound scaling method to obtain EfficientNet-B1 to B7.

The 3D transformation of this architecture is done by changing the 2D Convolutional layers to 3D convolutional layers, the 2D Depthwise Convolutional layers to 3D Depthwise Convolutional layers, the 2D Global Average Pooling layers to 3D Global Average Pooling layers, and by adjusting the 2 dimensional paddings to 3 dimensional paddings.

Table 2 presents the different architectures that we have been experimenting, by using different configurations for the width and depth factors, with the information of the initial and transformed input size. The 3D resolution adjustment follows the Equation (1)

(1) $$w_{3D}\times h_{3D}\times d_{3D}=w_{2D}\times h_{2D},$$

where $w_{3D}=h_{3D}=d_{3D}$ are, respectively, the width, height, and depth of the 3D input. All the three values are equal, since we want regular grids. $w_{2D}=h_{2D}$ are the width and height of the 2D resolution that Reference [18] proposes for EfficientNet, and which are are also equal. We name the 3D resolution $r_{3D}$ (Equation (2)) and the 2D resolution $r_{2D}$ (Equation (3)).

(2) $$r_{3D}=w_{3D}=h_{3D}=d_{3D},$$

(3) $$r_{2D}=w_{2D}=h_{2D}.$$

The transformation from 2D to 3D resolution expressed with Equation (1) can, therefore, be simplified as:

(4) $$r_{3D}=⎣\sqrt[{3}]{r_{2D}^{2}}⎦.$$

### 3.4. Classifier

The final layers of the Deep Neural Networks, or top of the network, depend on the purpose of the network. In our case, the network is aimed for classification. If the architecture has not its own top layers defined, the top is composed by a Fully Connected (FC) layer with a number of filters equal to the number of classes that we want to classify and a softmax activation layer. This softmax layer squashes a vector in the range [0,1], and the resulting elements add up to 1. During training a dropout layer is introduced before the FC layer.

## 4. Networks Training

All networks have been trained using a custom dataset composed of 7 industrial parts and 500 RGB-D images per object. The dataset is artificially generated using the Unreal Engine 4 (UE4) plugin NVIDIA Deep learning Dataset Synthesizer (NDDS) [33]. The creation of the dataset is described in Section 4.1.

We have trained all networks with stochastic gradient using Tensorflow [34]. We used Adam with a learning rate of 0.0001 in the first 20 epochs and 0.00001 in the last 10 epochs. We have only trained networks for 30 epochs to prevent network to saturate as the dataset is small. Since the aimed task is multi-class classification, in which there is only one element in the target vector which is not zero (the positive class), we use Categorical Cross-Entropy loss (Equation (5)). This loss is used because it is a very good measure of how distinguishable two discrete probability distributions are from each other.

(5) $$CE=-log\left(\frac{e^{s_{p}}}{\sum_{j}^{C}e^{s_{j}}}\right),$$

where $s_{p}$ is the score obtained from the net for the positive class.

### 4.1. Dataset generation

**Figure 10:**
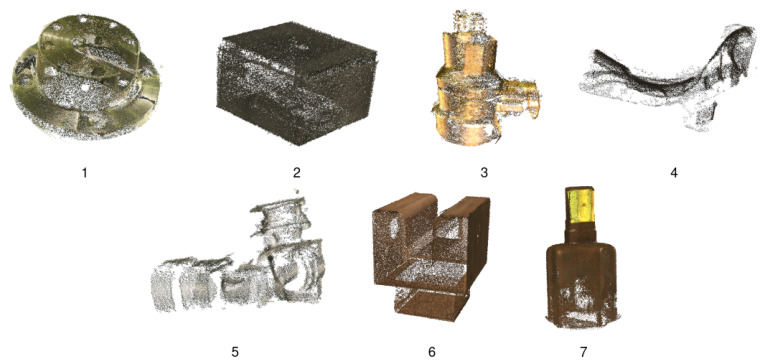
Reconstructed models of the 7 industrial parts.

As stated before, the dataset has been artificially generated using UE4 and NDDS plugin. All industrial parts are first reconstructed from point clouds obtained with high accuracy structured light sensor. Figure 10 shows captures of the reconstructed models of the 7 industrial parts. The obtained meshes are imported to UE4 and are randomly rotated and scaled, and they are rendered with a virtual RGB-D camera. For each image, we obtain the color image and the depth image, as well as a *json* file containing a set of relevant information, such as the 3D bounding box enclosing the object and its 3D location. For each object, 500 images are rendered, which produce a dataset of 3500 RGB-D images. This dataset is relatively small compared to other deep learning datasets. This is because the focus of this paper is to work with learning datasets of limited size as opposed to other methods in literature. Finally, the validation split is set to 10% of the instances of each class.

### 4.2. Data Preprocessing

The RGB-D image is first projected into a point cloud using the parameters of the camera used for capturing the RGB-D images in UE4. That point cloud is then segmented using the bounding box information and then discretized using a voxel grid. The size of the grid is different for each of the architectures. Table 3 shows the number of grids of each of the architectures used in this paper.

In order to avoid overfitting, different transformations are applied to the dataset. Each training sample is used unchanged, applied a flipping operation or rotated in any of its axis. This data augmentation introduces variability in the dataset in order to reduce the risk of bias.

### 4.3. Evaluation Metric

The aim of our proposal is multiclass classification, this is, each sample can only belong to one class. Therefore, the metric used for evaluating our model is categorical accuracy. This metric checks how many samples have been correctly labeled. To extend this evaluation, the precision (Equation (6)), the recall (Equation (7)), the F1 score (Equation (8)), and the macro-F1 (Equation (9)) of the highest accuracy model are going to be evaluated to analyze the behavior of the model for each class.

(6) $$precision_{i}=\frac{TP_{i}}{TP_{i}+FP_{i}},$$

(7) $$recall_{i}=\frac{TP_{i}}{TP_{i}+FN_{i}},$$

(8) $$F1_{i}=2\times \frac{precision_{i}\times recall_{i}}{precision_{i}+recall_{i}},$$

(9) $$\mathrm{macro}-F1=\frac{\sum_{i}^{C}F1_{i}}{\left|C\right|},$$

where $TP_{i}$ is the count of true positive instances of the class *i* (correctly classified instances), $FP_{i}$ is the count of false positive instances of the class *i* (the count of instances that are uncorrectly classified as class *i*), and $FN_{i}$ is the count of false negative instances of the class *i* (count of instances that should be classified as class *i* and they are classified as another class).

## 5. Experimental Results

The experiment was conducted using VGG16, ResNet, Inception ResNet v2 (from now on just Inception ResNet), and EfficientNet architectures. For each of the architectures, we have compared the results (i) without initializing the weights and (ii) using the pretrained weights from 2D architectures trained on Imagenet and transformed to 3D using extrusion and rotation.

For VGG16, results are not as good as expected. Therefore, no Figures are going to be included. The net classifies all instances the same class; therefore, the net is saturated. This happens for all the experiments (without weights, extrusion, and rotation).

**Figure 11:**
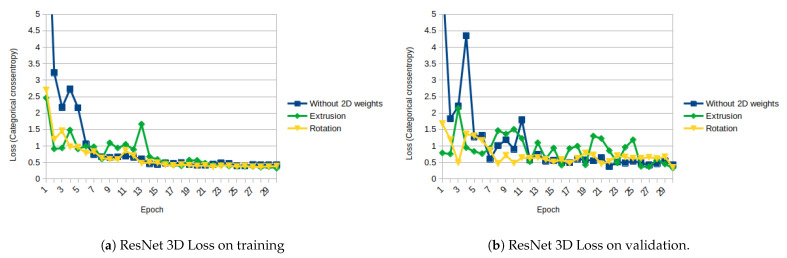
ResNet 3D Loss (Categorical Cross-Entropy).

**Figure 12:**
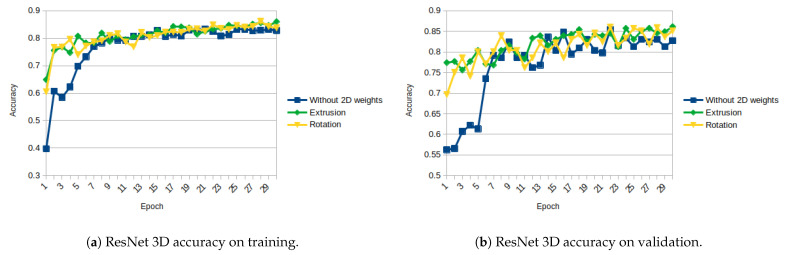
ResNet 3D accuracy.

For ResNet, the obtained results are more like expected. Figure 11a,b show the evolution of the losses using the 3 different weight initializations on training and on validation, respectively. The aformentioned figures show that initializing from 2D weights makes initial loss lower and converges earlier on both stages, training and validation. Regarding the accuracy, a similar behavior is observed: the initial and final accuracy are higher with the initialized weights. Figure 12a,b show the evolution of the accuracy on training and on validation, respectively.

**Figure 13:**
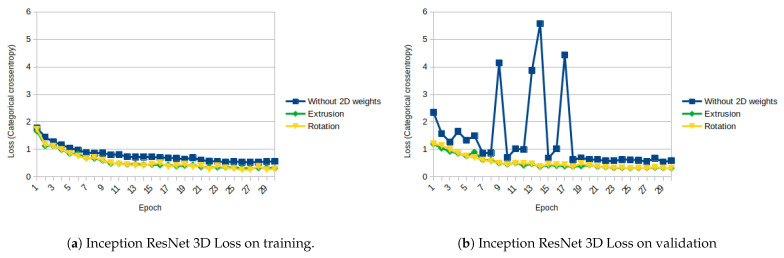
Inception ResNet 3D Loss (Categorical Cross-Entropy).

**Figure 14:**
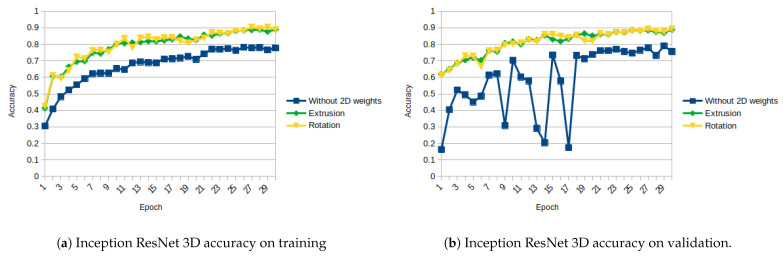
Inception ResNet 3D accuracy.

The results of Inception ResNet architecture are similar to the ones obtained with ResNet architecture, but the differences between the results obtained with the initialization and without initialization are even higher. Figure 13a,b and Figure 14a,b show, respectively, the evolution of the loss on training and validation, and the accuracy on training and validation.

Among the architectures tested, the Inception ResNet is the one that takes more profit from using the 2D weights, increasing its validation accuracy from 0.7558 to 0.8887 on extrusion and 0.8939 on rotation (increase of around 17 to 18%).

**Figure 15:**
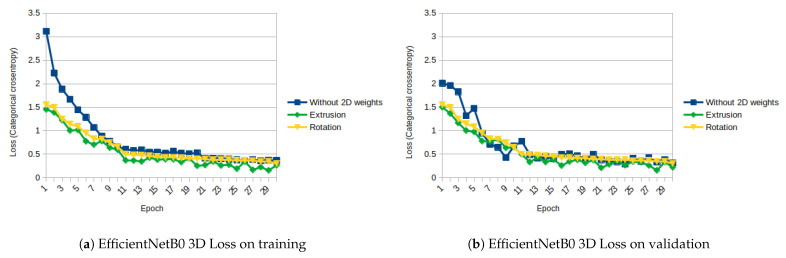
EfficientNetB0 3D Loss (Categorical Cross-Entropy).

**Figure 16:**
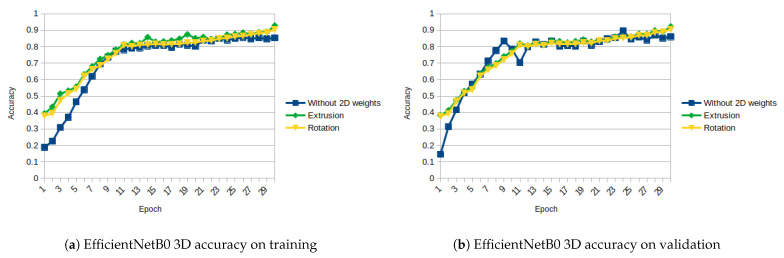
EfficientNetB0 3D accuracy.

EfficientNet is tested only from B0 up to B4 since our computing capabilities are limited to those set-ups. We observed that the performance of the network decreases from B0 to B4. This could be because the more complex the net is, the more epochs it needs to obtain similar results. This is even more evident in small datasets as the one we used. Figure 15a,b and Figure 16a,b show the evolution of the loss and accuracy only for B0 version, which performs better for our experimental set-up.

As shown in Figure 11, Figure 12, Figure 13, Figure 14, Figure 15 and Figure 16, almost in every epoch of the training of each model, the accuracy is higher using the initialized weights. For some of them, like the Inception ResNet, the difference of accuracy is notable.

**Figure 17:**
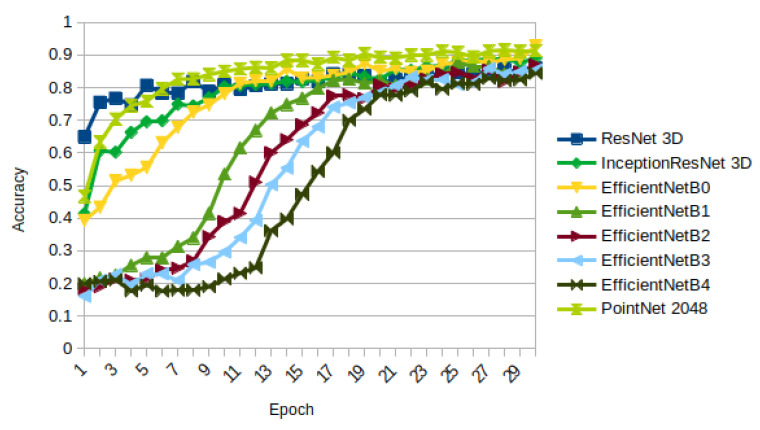
Accuracy on training: PointNet compared to all the architectures initialized with extrusion.

We compared all the extrusion architectures we propose with the PointNet [28], a 3D point-based deep learning method, by looking at the obtained accuracy during training (see Figure 17).

Table 4 shows the obtained results for all the architectures with all the weights initialized for the last epoch.

The highest accuracy is obtained with EfficientNetB0 initialized using extrusion (0.9276 in training and 0.9217 in validation). The largest improvement is achieved with Inception ResNet, increasing its accuracy from 0.7777 to 0.8901 and 0.8900 using extrusion and rotation, respectively, on training and from 0.7558 to 0.8887 and 0.8939 using extrusion and rotation, respectively, on validation.

To extend our analysis, we have calculated the confusion matrix of the EfficientNetB0 initialized using extrusion on validation since this is the architecture that obtains the highest accuracy. The confusion matrix is shown in Table 5. The network works similarly for every part, and the obtained Macro-F1 is really close to the accuracy. This is because the per-class precision and recall are very similar.

## 6. Conclusions

In this paper, we introduced a novel approach to transfer learned 2D convolutional networks to 3D convolutional networks in a reduced size dataset. We have studied several 2D architectures: VGG16, ResNet, Inception ResNet v2, and EfficientNet. The weights from pretrained 2D networks have been transformed to 3D using two approaches, the extrusion and the rotation.These transformed weights are then used to initialize the 3D version of those architectures.

On almost every architecture, the obtained accuracy is better with the 2D weights than without initialization, reaching a performance similar to state of art 3D deep learning methods.

In future works, other 2D to 3D transformations will be tested, looking for a better performance. Combinations of transformations may produce also improved results.

## Figures and Tables

**Table 1:** Architectures of ResNet.

Layer Group	Output Size	18-Layer	34-Layer	50-Layer	101-Layer	152-Layer
conv1	48×48×48	7×7×7, 64, stride 2				
conv2	24×24×24	3×3×3 max pool, stride 2				
		$\left[\begin{array}{c} 3\times 3\times 3,64\\ 3\times 3\times 3,64 \end{array}\right]\times 2$	$\left[\begin{array}{c} 3\times 3\times 3,64\\ 3\times 3\times 3,64 \end{array}\right]\times 3$	$\left[\begin{array}{c} 1\times 1\times 1,64\\ 3\times 3\times 3,64\\ 1\times 1\times 1,256 \end{array}\right]\times 3$	$\left[\begin{array}{c} 1\times 1\times 1,64\\ 3\times 3\times 3,64\\ 1\times 1\times 1,256 \end{array}\right]\times 3$	$\left[\begin{array}{c} 1\times 1\times 1,64\\ 3\times 3\times 3,64\\ 1\times 1\times 1,256 \end{array}\right]\times 3$
conv3	12×12×12	$\left[\begin{array}{c} 3\times 3\times 3,128\\ 3\times 3\times 3,128 \end{array}\right]\times 2$	$\left[\begin{array}{c} 3\times 3\times 3,128\\ 3\times 3\times 3,128 \end{array}\right]\times 4$	$\left[\begin{array}{c} 1\times 1\times 1,128\\ 3\times 3\times 3,128\\ 1\times 1\times 1,512 \end{array}\right]\times 4$	$\left[\begin{array}{c} 1\times 1\times 1,128\\ 3\times 3\times 3,128\\ 1\times 1\times 1,512 \end{array}\right]\times 4$	$\left[\begin{array}{c} 1\times 1\times 1,128\\ 3\times 3\times 3,128\\ 1\times 1\times 1,512 \end{array}\right]\times 8$
conv4	6×6×6	$\left[\begin{array}{c} 3\times 3\times 3,256\\ 3\times 3\times 3,256 \end{array}\right]\times 2$	$\left[\begin{array}{c} 3\times 3\times 3,256\\ 3\times 3\times 3,256 \end{array}\right]\times 6$	$\left[\begin{array}{c} 1\times 1\times 1,256\\ 3\times 3\times 3,256\\ 1\times 1\times 1,1024 \end{array}\right]\times 6$	$\left[\begin{array}{c} 1\times 1\times 1,256\\ 3\times 3\times 3,256\\ 1\times 1\times 1,1024 \end{array}\right]\times 23$	$\left[\begin{array}{c} 1\times 1\times 1,256\\ 3\times 3\times 3,256\\ 1\times 1\times 1,1024 \end{array}\right]\times 36$
conv5	3×3×3	$\left[\begin{array}{c} 3\times 3\times 3,512\\ 3\times 3\times 3,512 \end{array}\right]\times 2$	$\left[\begin{array}{c} 3\times 3\times 3,512\\ 3\times 3\times 3,512 \end{array}\right]\times 3$	$\left[\begin{array}{c} 1\times 1\times 1,512\\ 3\times 3\times 3,512\\ 1\times 1\times 1,2048 \end{array}\right]\times 3$	$\left[\begin{array}{c} 1\times 1\times 1,512\\ 3\times 3\times 3,512\\ 1\times 1\times 1,2048 \end{array}\right]\times 3$	$\left[\begin{array}{c} 1\times 1\times 1,512\\ 3\times 3\times 3,512\\ 1\times 1\times 1,2048 \end{array}\right]\times 3$
	1×1×1	average pool				

**Table 2:** Parameters of each of the EfficientNet architectures (width factor, depth factor, 2D resolution, and 3D resolution).

EfficientNet Architecture	w	d	$r_{2D}$	$r_{3D}$
B0	1.0	1.0	224	36
B1	1.0	1.1	240	38
B2	1.1	1.2	260	40
B3	1.2	1.4	300	44
B4	1.4	1.8	380	52
B5	1.6	2.2	456	60
B6	1.8	2.6	528	66
B7	2.0	3.1	600	72

**Table 3:** Number of grids of each architecture used by the Voxel grid.

Architecture	Number of Grids
VGG16	96 × 96 × 96
ResNet	96 × 96 × 96
Inception-ResNet v2	139 × 139 × 139
EfficientNet B0	36 × 36 × 36
EfficientNet B1	38 × 38 × 38
EfficientNet B2	40 × 40 × 40
EfficientNet B3	44 × 44 × 44
EfficientNet B4	52 × 52 × 52
EfficientNet B5	60 × 60 × 60
EfficientNet B6	66 × 66 × 66
EfficientNet B7	72 × 72 × 72

**Table 4:** Accuracy and loss comparative between all the used architectures and PointNet. Last column is the number of parameters each network has to train.

Method	Train		Val		Params
	Loss	acc	Loss	acc	
ResNet 3D	0.4284	0.8279	0.4279	0.8272	47M
ResNet 3D Extrusion	0.3245	0.8599	0.3312	0.8612	
ResNet 3D Rotation	0.3892	0.8372	0.3484	0.8512	
Inception ResNet 3D	0.5653	0.7777	0.592	0.7558	67M
Inception ResNet 3D Extrusion	0.3127	0.8901	0.3157	0.8887	
Inception ResNet 3D Rotation	0.2880	0.8900	0.3133	0.8939	
EfficientNetB0 3D	0.3664	0.8534	0.3137	0.8605	4.7M
EfficientNetB0 3D Extrusion	0.2663	0.9276	0.2213	0.9217	
EfficientNetB0 3D Rotation	0.0312	0.9039	0.3004	0.9052	
EfficientNetB1 3D	0.3987	0.847	0.367	0.8372	7.5M
EfficientNetB1 3D Extrusion	0.3758	0.8771	0.3610	0.8420	
EfficientNetB1 3D Rotation	0.2325	0.8485	0.3526	0.8422	
EfficientNetB2 3D	0.4393	0.8324	0.3408	0.8547	8.8M
EfficientNetB2 3D Extrusion	0.3880	0.8592	0.3384	0.8595	
EfficientNetB2 3D Rotation	0.3273	0.8362	0.3271	0.8549	
EfficientNetB3 3D	0.459	0.8289	0.3906	0.8285	12.1M
EfficientNetB3 3D Extrusion	0.3887	0.8422	0.3809	0.8305	
EfficientNetB3 3D Rotation	0.4111	0.8583	0.3856	0.8318	
EfficientNetB4 3D	0.4785	0.8176	0.4106	0.8097	19.7M
EfficientNetB4 3D Extrusion	0.3960	0.8544	0.3949	0.8123	
EfficientNetB4 3D Rotation	0.4335	0.8207	0.4039	0.8153	
PointNet	1.1802	0.9132	1.2587	0.9048	1M

**Table 5:** Confusion matrix and metrics of EfficientNetB0 initialized using extrusion.

		Real Class							Total
		1	2	3	4	5	6	7	
Predicted class	1	486	1	0	20	1	10	3	
	2	7	492	0	11	5	5	2	
	3	2	7	443	1	45	3	20	
	4	5	0	1	455	2	0	2	
	5	0	0	55	3	447	0	0	
	6	0	0	1	3	0	472	42	
	7	0	0	0	7	0	10	431	
Precision		0.972	0.984	0.886	0.91	0.894	0.944	0.862	
Recall		0.933	0.943	0.85	0.978	0.885	0.911	0.962	
F1		0.952	0963	0.868	0.943	0.89	0.927	0.909	
Macro-F1									0.922
Accuracy									0.9217

## Data Availability

The data presented in this study are available on demand from thecorresponding author.
